# Bias in AI systems: integrating formal and socio-technical approaches

**DOI:** 10.3389/fdata.2025.1686452

**Published:** 2026-01-08

**Authors:** Amar Ahmad, Yvonne Vallès, Youssef Idaghdour

**Affiliations:** Public Health Research Center, New York University Abu Dhabi, Abu Dhabi, United Arab Emirates

**Keywords:** algorithmic bias, fairness in machine learning, ethical AI, responsible AI, bias mitigation, socio-technical systems

## Abstract

Artificial Intelligence (AI) systems are increasingly embedded in high-stakes decision-making across domains such as healthcare, finance, criminal justice, and employment. Evidence has been accumulated showing that these systems can reproduce and amplify structural inequities, leading to ethical, social, and technical concerns. In this review, formal mathematical definitions of bias are integrated with socio-technical perspectives to examine its origins, manifestations, and impacts. Bias is categorized into four interrelated families: historical/representational, selection/measurement, algorithmic/optimization, and feedback/emergent, and its operation is illustrated through case studies in facial recognition, large language models, credit scoring, healthcare, employment, and criminal justice. Current mitigation strategies are critically evaluated, including dataset diversification, fairness-aware modeling, post-deployment auditing, regulatory frameworks, and participatory design. An integrated framework is proposed in which statistical diagnostics are coupled with governance mechanisms to enable bias mitigation across the entire AI lifecycle. By bridging technical precision with sociological insight, guidance is offered for the development of AI systems that are equitable, accountable, and responsive to the needs of diverse populations.

## 1 Introduction

Artificial intelligence (AI) technologies now play a central role in shaping decisions in domains such as healthcare, criminal justice, finance, education, and employment. While these systems can improve efficiency and scale, growing evidence shows that they often reflect and reinforce existing societal inequalities (Mehrabi et al., 2021; Lacmanovic and Skare, 2025). As AI models, especially those based on deep learning, become more complex and difficult to interpret, the need to understand and address bias in their development and deployment becomes increasingly urgent (Raji et al., 2022; Ferrara, 2024).

### 1.1 Audience and scope

This manuscript is submitted as a *Mini Review*. It draws on 72 published sources and is intended as a tutorial synthesis for an interdisciplinary readership of machine-learning practitioners, statisticians, and AI-policy researchers. While we reference global governance frameworks to motivate relevance, our primary contribution is technical: we provide a formal bias decomposition and illustrate its use in practical lending and health-care contexts. Proposition 2, Lemma 1, and Corollaries 1–2 restate foundational results for didactic clarity and do not introduce new theoretical claims or models.

With the advancement of deep-learning techniques, the concern over bias, whether in the creation or execution of an AI model, grows as well. What was once a theoretical concern has become a practical and policy-relevant issue. Regulatory and ethical frameworks are emerging globally, such as the OECD AI Principles (OECD, 2019) and the U.S. Blueprint for an AI Bill of Rights (White House OSTP, 2022). Investigative journalism has also brought attention to this issue. For instance, ProPublica's 2016 report revealed racial bias in criminal risk assessment tools (Angwin et al., 2016), while the Gender Shades study exposed major disparities in facial-recognition performance for darker-skinned women (Buolamwini and Gebru, 2018). These cases illustrate why addressing AI bias requires an interdisciplinary approach, combining insights from computer science, law, ethics, and the social sciences (Selbst et al., 2019; Crawford, 2021).

In a study conducted across five U.S. metropolitan areas, Koenecke et al. (2020) reported that commercial speech-recognition systems produced roughly twice the word-error rate for speakers who self-identify as Black.

Likewise, Obermeyer et al. (2019), using claims data from a large U.S. insurer, found that a widely deployed health-risk score systematically underestimated the needs of Black patients relative to White patients with comparable disease burden.

Concerns about bias in algorithmic systems date back to the 1980s and 1990s, when early expert systems were already found to behave in discriminatory ways (Danks and London, 2017). But those warnings were largely overlooked. In the 2010s, as machine learning became widely adopted in high-stakes domains, algorithmic harms drew broader attention (Barocas et al., 2019). Landmark investigations, such as ProPublica's analysis of the COMPAS tool (Angwin et al., 2016) and Buolamwini and Gebru's Gender Shades study (Buolamwini and Gebru, 2018), catalyzed public concern and academic inquiry. These developments paved the way for global debates about accountability, transparency, and fairness in AI (Hardt et al., 2016; Hooker, 2021; Kleinberg et al., 2019; Perra and Rocha, 2019; Rothschild and Stiglitz, 1970).

### 1.2 External validity and geographic scope

Most large-scale bias studies rely on datasets from the United States or Western Europe. The magnitude, and sometimes even the direction, of algorithmic bias can vary across jurisdictions because protected attributes (race, caste, socio-economic status, dialect) intersect with local histories of marginalization (Birhane et al., 2022; Abebe et al., 2020). Results drawn from U.S. data, such as (Koenecke et al., 2020; Obermeyer et al., 2019), therefore must not be assumed to generalize to all global Black populations; accent, dialect or income may be the operative factors elsewhere. We flag this limitation to motivate cross-regional audits and the study of low-resource fringe cases where bias often goes unnoticed (Barocas and Selbst, 2016; Liu et al., 2018). Our aim is therefore didactic rather than exhaustive, and we make no claim to systematic coverage of the entire literature.

**Figure 1:**
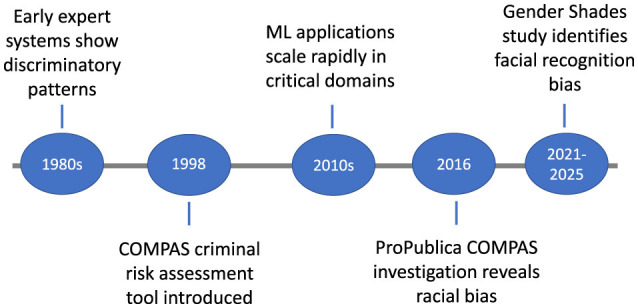
Timeline of key milestones in the evolution of awareness and regulatory responses to algorithmic bias, spanning from early technical developments in the 1980s to global policy initiatives introduced between 2021 and 2025.

Figure 1 traces the evolution of algorithmic bias, highlighting major milestones from the 1980s to recent regulatory initiatives introduced between 2021 and 2025. The timeline begins with early expert systems in the 1980s, which exhibited discriminatory patterns, and progresses to the introduction of the COMPAS criminal risk assessment tool in 1998.

It further charts the rapid scaling of ML applications in critical domains during the 2010s, the 2016 ProPublica investigation exposing racial bias in COMPAS, and the 2021–2025 period, marked by the Gender Shades study and emerging policy measures addressing bias in AI systems, spanning racial, gender, socioeconomic, and linguistic dimensions. These milestones underscore the growing recognition of algorithmic bias and motivate the need for a systematic taxonomy of its forms. Accordingly, all subsequent sections are organized around four analytically distinct but practically intertwined families of bias. Historical or representational bias originates in unequal social power relations that become encoded in textual or visual corpora; evidence for its presence has usually been derived from dataset audits or embedding association tests, quantified for example by the Word Embedding Association Test (WEAT) effect size. Selection and measurement bias arise when marginalized groups are systematically omitted or mislabeled during data collection; such bias has been detected through missingness heat maps, label-flip analyses, and the selection ratio *R*_sel_ in Equation 4.

Algorithmic or optimisation bias arises when empirical risk minimization is carried out without any fairness constraint; its magnitude can be measured with group-fairness metrics such as *equalized odds* (defined later in Equation 14) or with the amplification ratio α(*f*_θ_) (introduced in Equation 7). Finally, *feedback* or *emergent* bias occurs once model outputs influence future inputs, leading to self-reinforcing disparities that are analyzed via the dynamical-systems framework laid out in Equations 8–10.

This paper analyzes the origins of AI bias by looking at both technical and sociotechnical factors. It examines how biases in data, model design, and feedback loops lead to real-world harms. The paper also assesses current strategies for mitigating these harms, ranging from dataset design to regulatory oversight, and proposes an integrated framework for building more equitable AI systems.

### 1.3 Clarifying scope, definitions, and evidence base

A clear foundation for the discussion that follows is provided in this subsection by clarifying the scope of the mini-review, stating the definition of bias adopted in the manuscript, and outlining the measurement procedures employed in the cited studies.

**Operational Definition**: A learning system is regarded as *biased* if, for some protected attribute *A* such as race, gender, or disability, at least one widely accepted fairness metric reports a non-zero disparity between the predicted outcome Ŷ and the ground-truth label *Y*. Formally,


$$\begin{array}{l} \exists F\text{s.t.}F\left(Ŷ,Y,A\right)>0, \end{array}$$ (1)

where *F* may instantiate a group fairness statistic (for example, ΔTPR, ΔFPR, or ΔRisk in Equation 12), an individual fairness distance, or a causal counterfactual divergence.

Bias is categorized into four interrelated families: historical or representational bias, selection or measurement bias, algorithmic or optimization bias, and feedback or emergent bias. Each category is characterized by distinct mechanisms through which unfairness can be introduced or reinforced, and their manifestations are illustrated in this mini-review through case studies in facial recognition, large language models, credit scoring, healthcare, employment, and criminal justice.

Although race and gender provide the most thoroughly documented examples in the current literature, the analytic framework we employ extends to disability status, age, caste, dialect, religious affiliation, and their many intersections (Hanna et al., 2020; Holstein et al., 2019).

In more practical terms, a learning system is considered biased whenever, for a protected attribute such as race, gender, or disability, any widely recognized fairness test reveals a difference between what the model predicts and what actually occurs. The fairness test can be: (i) *Group fairness*, which checks, for example, whether one group receives a higher false-positive rate or lower true-positive rate than another. (ii) *Individual fairness*, which asks whether similar individuals are treated similarly. (iii) *Counterfactual fairness*, which asks whether the decision would change if only the protected attribute were changed.

This single rule of thumb provides a unifying criterion that accommodates the heterogeneous fairness notions encountered across disciplines, enabling discussions about bias that span different research communities.

Bias is evaluated at three successive stages of the machine-learning pipeline. During *dataset development* the divergence *B*_data_ (Equation 2), the selection ratio *R*_sel_ (Equation 4), and the label-bias statistic *B*_label_ (Equation 5) are computed to identify structural skews before any model training commences. During *training* the primary task loss is optimized subject to fairness constraints of the form Equation 15 or fairness penalties of the form Equation 16. During *deployment* the closed-loop system is continuously monitored for drift in disparity measures and for instabilities indicated by a spectral radius ρ(*J*_*h*_)>1 (Equation 10).

Even when an investigation focuses on a single bias type, the remaining types often surface implicitly. For instance, debiasing word embeddings tends to alter class priors, which can re-introduce selection bias at train-test time. *Post-hoc* threshold adjustment may satisfy a chosen fairness constraint momentarily, but feedback dynamics can erode the gains once the model interacts with the world. Contemporary regulatory instruments, exemplified by the OECD AI Principles and the NIST AI Risk Management Framework, are already mandating end-to-end artifact tracing that spans data, model, and deployment environments. A panoramic view therefore remains indispensable, even if subsequent research narrows its empirical scope.

No claim is made that the metrics highlighted here exhaust the space of fairness diagnostics, nor that the documented case studies form a complete catalog of algorithmic harm. The intention is rather to provide a precise formal substrate, together with clearly referenced empirical findings, such that future work can select, refine, or discard elements as appropriate for narrower research questions. In this way the mini-review balances breadth with definitional and evidentiary clarity, thereby addressing the main concern articulated in the feedback.

#### 1.3.1 TTP (technical, technical-policy-aware)

We make three tightly coupled contributions aimed at both method builders and regulation-minded auditors: (i) a formal decomposition of algorithmic bias (Lemma 1) that cleanly separates data imbalance from model capacity; (ii) two corollaries that transform the decomposition into domain-agnostic mitigation rules ready for turnkey use in credit-scoring pipelines; and (iii) an explicit mapping of those rules onto current legal obligations, including EU AI Act Articles 10 & 15 and U.S. ECOA/CFPB guidance, thereby showing how practitioners can satisfy technical performance targets and policy compliance within one unified workflow.

As outlined above, bias can be understood through four interrelated families-historical or representational, selection or measurement, algorithmic or optimization, and feedback or emergent. Each operates through distinct mechanisms by which unfairness can be introduced or reinforced across the AI lifecycle. Their manifestations are illustrated in this mini-review through case studies spanning facial recognition, large language models, credit scoring, healthcare, employment, and criminal justice.

### 1.4 Bias beyond supervised learning

Most case studies discussed so far involve *supervised* learning, where bias is measured as a disparity between labels and predictions. Two other paradigms, unsupervised representation learning and modern generative models, exhibit related but distinct bias mechanisms.

#### 1.4.1 Unsupervised pipelines

Because no ground-truth labels exist, bias manifests in the geometry of the learned embedding space or in cluster-membership decisions. Empirical studies show that sociodemographic groups may form separable sub-manifolds, enabling downstream tasks to inherit implicit group tags Li et al. (2020); Jaiswal et al. (2018). Mitigation therefore targets the representation itself (e.g. adversarial invariance, fair PCA) rather than confusion-matrix gaps.

#### 1.4.2 Generative AI

Large language and diffusion models sample from an implicit distribution *p*_θ_(*y*∣*x*). Hallucinations correspond to low-density outliers, whereas demographic stereotypes correspond to a *mean shift* $∥\mu_{\theta }-\mu^{⋆}∥>0$ relative to an externally specified ground-truth mean μ^⋆^ Ji et al. (2023); Bender et al. (2021). Both phenomena fall under our *feedback/emergent* family because they arise after repeated model, user interaction. Debiasing techniques include distribution calibration, rejection sampling, and reinforcement learning from human feedback Ji et al. (2023).

In summary, supervised, unsupervised, and generative settings share common root causes, skewed data, optimization objectives, and feedback loops, but the *measurement locus* of bias shifts from label disparity (supervised) to representation geometry (unsupervised) to distribution shift (generative).

#### 1.4.3 Literature-selection rationale

The 72 references cited in this Mini Review were originally curated as core readings for the undergraduate course *AI and Human Decisions* (New York University Abu Dhabi, 2025). They were retained because each either (i) presents well-documented empirical evidence of one of the four bias families introduced below, or (ii) describes a mitigation technique that has been independently reproduced in at least one applied domain. Our goal is therefore pedagogical rather than exhaustive, and we make no claim to systematic coverage of the full literature.

#### 1.4.4 Road-map

Section 2 categorizes different types of bias found in AI systems. Section 3 presents real-world examples across several domains. Section 4 reviews current mitigation strategies. Section 6 reflects on open challenges, and Section 7 summarizes the main findings. Supplementary material appears online.

## 2 Types of bias in AI

Biases in training data are among the most thoroughly documented sources of algorithmic unfairness (Mehrabi et al., 2021; Lacmanovic and Skare, 2025). As AI systems are increasingly adopted in sensitive areas such as healthcare, finance, and criminal justice, concerns about fairness have intensified. A growing body of research shows that these technologies often reproduce and even exacerbate existing social inequalities.

Algorithmic biases in health care arise through three main pathways. First, these biases often reflect the persistence of historical inequities embedded in legacy datasets, which encode disparities in access to care and treatment. Second, they can result from the reliance on flawed proxies, such as healthcare costs being used as a substitute for health needs. As a study (Obermeyer et al., 2019) demonstrates, this approach disproportionately underestimates the health needs of Black patients, as less money is spent on their care despite similar levels of illness compared to White patients. Finally, even data that appears objective can perpetuate and amplify social stratification, particularly when learning algorithms emphasize correlations that mirror existing systemic inequities. Addressing these sources of bias, such as reformulating proxies, is critical to improving fairness and equity in predictive health care systems.

Selection bias introduced during data collection is another significant source of algorithmic unfairness. Datasets often inherit the prejudices of previous decision-makers or reflect structural inequalities in society at large (Barocas and Selbst, 2016). For instance, individuals from historically disadvantaged groups may be underrepresented in the data or misrepresented due to lower data quality, stemming from limited access to services, technological barriers, or biased institutional practices. These gaps are rarely random: marginalized communities are more likely to reside in data shadows, leading to their systematic omission from predictive models. Such omissions are difficult to detect and even harder to correct, especially when these biases are normalized within routine data workflows. The result is a feedback loop where historical exclusion is formalized into seemingly objective algorithmic decisions. Furthermore, measurement disparities also play a role (Chen and Hooker, 2023). When model optimization prioritizes overall accuracy without regard to group-specific performance, predictive outcomes can vary substantially across demographic groups. For example, instruments calibrated for majority populations may systematically underperform for marginalized groups, further entrenching disparities.

When machine learning models are trained on biased or incomplete data, they often internalize these patterns and treat them as predictive features. This is evident in employment data, where long-standing gender wage gaps, estimated between 17% and 21%, persist (Blau and Kahn, 2017). Similarly, in the U.S. mortgage market, Black and Latinx borrowers were found to pay between 5.4 and 7.7 basis points more than White borrowers with comparable credit risk, with disparities rising to 13.8 basis points in predominantly minority neighborhoods (Bartlett et al., 2022). These examples underscore how algorithmic systems, when left unchecked, can reinforce deeply rooted social inequities.

Algorithmic decision-making can obscure responsibility for discriminatory outcomes by presenting them as the product of neutral computation rather than human or institutional bias (Barocas and Selbst, 2016) observed. Such biases may arise from statistically valid but socially harmful patterns, which can reinforce historical inequalities (Selbst et al., 2019). Because these effects lack the transparency of explicit discrimination, they are often more difficult to detect, interpret, or contest.

### 2.1 Mathematical formalization

The gap between the training distribution *P*_train_ and the target (population) distribution *P*_pop_ can be quantified with the Kullback-Leibler (KL) divergence


$$\begin{array}{l} B_{\text{data}}=D_{\text{KL}}\left(P_{\text{train}}||P_{\text{pop}}\right)={𝔼}_{x\sim P_{\text{train}}}\left[\log\frac{P_{\text{train}}\left(x\right)}{P_{\text{pop}}\left(x\right)}\right], \end{array}$$ (2)

which is finite whenever *P*_train_≪*P*_pop_.

Let the binary variable *S*∈{0, 1} indicate whether an individual is selected into the data set. The observed density is


$$\begin{array}{l} P_{\text{train}}\left(x\right)=P_{\text{pop}}\left(x|S=1\right)\neq P_{\text{pop}}\left(x\right), \end{array}$$ (3)

so the *selection ratio*


$$\begin{array}{l} R_{\text{sel}}\left(x\right)=\frac{P_{\text{train}}\left(x\right)}{P_{\text{pop}}\left(x\right)}\;(\text{defined only where }P_{\text{pop}}\left(x\right)>0\text{)} \end{array}$$ (4)

identifies over- (*R*_sel_>1) and under-sampled regions.

Let Y^*^ denote the ideal (error-free) label and *Y* the observed label. For a protected attribute value *A* = *a* we define


$$\begin{array}{l} B_{\text{label}}\left(a\right)={𝔼}_{X|A=a}\left[P\left(Y^{*}=1|X,A=a\right)-P\left(Y=1|X,A=a\right)\right], \end{array}$$ (5)

measured in *percentage points* (difference of Bernoulli means).

Appendix 8.1 provides a fully worked numerical example on the UCI German-Credit data set (Hugging Face mirror), including the empirical audit results, *B*_data_ = 0.067 nats, *R*_sel_ = 0.90, and *B*_label_ = −7.5 pp, that illustrate every step of the calculation pipeline.

### 2.2 Amplification mechanisms

Given a model *f*_θ_ with parameters θ, empirical risk minimization (ERM) solves


$$\begin{array}{l} \underset{\theta }{\min}\left\{{𝔼}_{\left(x,y\right)\sim P_{\text{train}}}\left[L\left(f_{\theta }\left(x\right),y\right)\right]+\lambda \text{ Ω}\left(\theta \right)\right\}, \end{array}$$ (6)

where L is the task loss and Ω a regulariser.

Let *D*(·||·) be a divergence (we use KL for both numerator and denominator). For any two input distributions $P,P^{\prime }\in P$ that are absolutely continuous w.r.t. a common base measure, define


$$\begin{array}{l} \alpha \left(f_{\theta }\right)=\underset{P,P^{\prime }\in P}{\sup}\frac{D_{\text{KL}}\left(f_{\theta }\left(P\right)||f_{\theta }\left(P^{\prime }\right)\right)}{D_{\text{KL}}\left(P||P^{\prime }\right)}. \end{array}$$ (7)

α(*f*_θ_)>1 indicates that the model magnifies distributional differences present in the data.

### 2.3 Dynamical-Systems perspective

Let the system state be ${\text{s}}_{t}\in {\mathbb{R}}^{d}$ and the algorithmic action ${\text{a}}_{t}\in {\mathbb{R}}^{m}$. A generic feedback system is


$$\begin{array}{l} {\text{s}}_{t+1}=f\left({\text{s}}_{t},{\text{a}}_{t}\right),\;{\text{a}}_{t}=g\left({\text{s}}_{t},\beta \right), \end{array}$$ (8)

with policy parameter β. Eliminating **a**_*t*_ gives the *closed-loop* map *h*(**s**) = *f*(**s**, *g*(**s**, β)). Its Jacobian is


$$\begin{array}{l} J_{h}=\underset{J_{f}^{\left(s\right)}}{\underset{︸}{\frac{\partial f}{\partial \text{s}}}}+\underset{J_{f}^{\left(a\right)}}{\underset{︸}{\frac{\partial f}{\partial \text{a}}}}\underset{J_{g}}{\underset{︸}{\frac{\partial g}{\partial \text{s}}}}, \end{array}$$ (9)

and the system is (linearly) unstable when


$$\begin{array}{l} \rho \left(J_{h}\right)>1, \end{array}$$ (10)

where ρ(·) denotes the spectral radius.

Let *p*_*t*_(*x*) be the predicted crime probability at location x∈X and time *t*, and let *c*_*t*_(*x*)≥0 be the observed crime count. A simple feedback update is


$$\begin{array}{l} p_{t+1}\left(x\right)=\left(1-\gamma \right)p_{t}\left(x\right)+\gamma \frac{c_{t}\left(x\right)}{\int_{X}c_{t}\left(u\right)du},\;\gamma \in \left[0,1\right]. \end{array}$$ (11)

The normalizing denominator ensures $\underset{X}{\int }p_{t+1}\left(u\right)du=1$. When the closed-loop spectral radius (Equation 10) exceeds 1, small spatial perturbations–often reflecting historical over-policing–grow exponentially, entrenching bias; for ρ(*J*_*h*_) < 1 they decay.

Together, Equations 2–11 provide a self-consistent mathematical framework for analyzing how statistical biases arise, propagate through learning objectives, and are amplified by real-world feedback.

**Figure 2:**
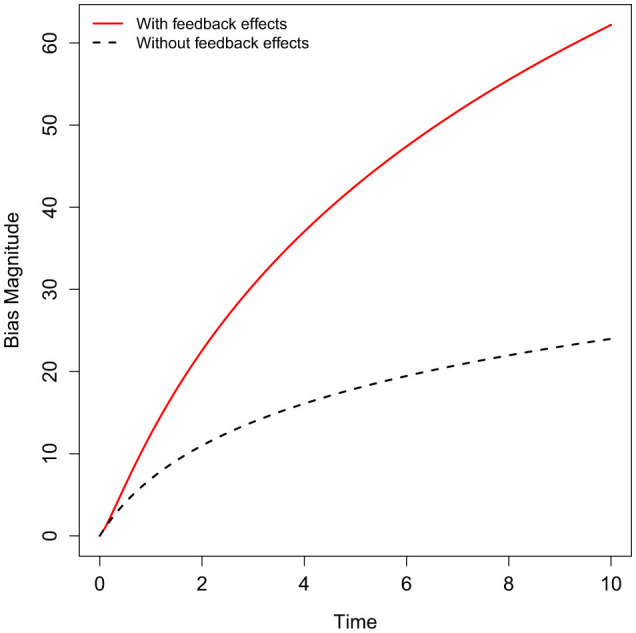
Dynamical evolution of bias magnitude over time, contrasting systems with feedback effects (ρ < 1, red solid line) and those without such effects (ρ < 1, dark-blue dashed line). Feedback loops can amplify initial disparities, leading to compounding increases in bias over time, whereas stable systems show slower growth that eventually plateaus. This conceptual illustration underscores the importance of accounting for feedback dynamics when assessing long-term fairness impacts in real-world AI deployments. The axes depict abstract time progression and relative bias magnitude, and do not represent empirical measurements.

Figure 2 illustrates the evolution of bias magnitude over time in algorithmic systems, comparing scenarios with and without feedback effects. In systems influenced by feedback loops (red curve, ρ>1), initial disparities are amplified by the model's outputs, resulting in a compounding increase in bias over time. This dynamic mirrors real-world contexts such as predictive policing or credit scoring, where model predictions shape future data collection and institutional responses, creating a self-reinforcing cycle of bias. In contrast, the black dashed line represents a stable system without feedback effects (e.g., ρ < 1), where bias levels increase slowly and eventually plateau. This comparison highlights the critical role of feedback loops in exacerbating bias and underscores the importance of incorporating these dynamics into fairness assessments. Ignoring feedback effects can lead to a significant underestimation of the long-term societal harms posed by biased AI systems, emphasizing the need for proactive interventions to disrupt these cycles.

### 2.4 Emerging dimensions of bias in AI

Traditional categorisations of bias, namely data, algorithmic, and representational bias, have been extended to incorporate several emerging dimensions that reflect recent developments in artificial intelligence.

Generative models, including large language and multimodal systems, are often found to produce hallucinated content-outputs that sound fluent but contain incorrect or misleading information. These hallucinations are especially likely when the input is ambiguous, and they become more serious in sensitive fields such as medicine or law (Huang et al., 2025). This issue, sometimes referred to as semantic drift, has been studied as a gap between how natural the language sounds and how accurate the facts are. In addition, it has been shown that generative systems trained on large datasets can pick up and repeat social stereotypes. These biases, which are already present in the data, can be amplified in both text and image generation tasks (Huang et al., 2025). As a result, stereotypes about gender or race may be reinforced without being directly programmed into the models.

Recent evidence from medical AI applications shows that multimodal foundation models, such as vision-language architectures, encode and amplify demographic biases across modalities. For instance, state-of-the-art chest X-ray models have been shown to underdiagnose historically marginalized subgroups, including Black female patients, despite their apparent expert-level performance (Seyyed-Kalantari et al., 2021).

Multimodal models that combine data sources such as images, structured clinical records, and time-series signals often outperform unimodal systems in predictive tasks. While these performance gains are well documented, fairness outcomes tend to vary across subgroups. Adding new modalities during training has been shown to improve overall accuracy, yet disparities in fairness metrics-such as true positive rates and demographic parity-can persist or even increase depending on the evaluation setting (Sampath et al., 2025).

Missing modalities at inference time further complicate deployment. When inputs are incomplete, model performance declines, and fairness across demographic groups is compromised. This sensitivity to data availability raises concerns about the robustness of multimodal AI systems in high-stakes environments, particularly in healthcare. The findings emphasize the need to evaluate multimodal models beyond accuracy, with equal attention to equity and reliability (Sampath et al., 2025).

Temporal bias has been recognized in sequential decision-making systems, including reinforcement learning and adaptive testing frameworks. In such contexts, fairness-related challenges have been found to differ substantially from those encountered in static classification tasks. While most existing correction approaches disregard temporal dependencies, recent work has demonstrated that attention-based probabilistic models can be effectively employed to correct for long-range temporal patterns (Nivron et al., 2025). Their method re-frames bias correction as a probabilistic modeling task, yielding more accurate adjustments in sequential data and offering promising implications for fairness in time-dependent machine learning applications.

## 3 Examples of bias in AI

### 3.1 Facial recognition systems

Facial recognition technologies remain among the most visible and critically examined domains for algorithmic bias. Seminal work by Buolamwini and Gebru (2018) revealed stark disparities in gender classification accuracy across demographic groups, with commercial systems exhibiting error rate gaps exceeding 30 percentage points between lighter-skinned males and darker-skinned females. These disparities stem from imbalanced training data, underrepresentation of non-White subgroups, and evaluation practices that often fail to account for intersectional fairness. Although some technical improvements have been reported in subsequent audits, systemic bias persists, particularly when model performance is reported in aggregate rather than by subgroup (Raji et al., 2022). This emphasizes that commercial benchmarks often obscure disproportionate harms by failing to disaggregate performance data, thereby enabling biased systems to appear more equitable than they are in practice.

Table 1 summarizes gender classification error rates reported for three commercial systems Microsoft (*MSFT*), *Face*++, and *IBM*, across four demographic subgroups (Buolamwini and Gebru, 2018). Error rates are averaged across the Pilot Parliaments Benchmark (PPB) and its South African subset. While *MSFT* shows lower absolute error rates than the other systems, it still exhibits substantial disparities: the average error for darker-skinned females (22.3%) remains over 22 percentage points higher than for lighter-skinned males (0.0%). All three classifiers share this pattern of intersectional bias, consistently performing worst on darker-skinned females and best on lighter-skinned males. These disparities persist despite differences in overall accuracy, underscoring that lower error rates do not imply fairness. Given the opacity of commercial model development pipelines, it is unclear whether these differences reflect inclusive training data, threshold tuning, or optimizations favoring majority groups.

**Table 1:** Average gender classification error rates across the PPB and South African datasets for each demographic group and vendor, based on Buolamwini and Gebru (2018).

**Demographic group**	***MSFT***	***Face*++**	***IBM***
*Darker-skinned females*	22.3%	35.3%	33.9%
*Darker-skinned males*	3.0%	0.6%	8.9%
*Lighter-skinned females*	0.9%	8.6%	3.6%
*Lighter-skinned males*	0.0%	0.4%	4.3%

Most widely used facial analysis datasets, such as *LFWA*+, *CelebA*, *COCO*, and *IMDB*−*WIKI*, exhibit extreme overrepresentation of White individuals, with only minimal inclusion of non-White subgroups. This imbalance in training and benchmark datasets contributes to the persistent disparities in model performance across racial and ethnic groups. In response to these limitations, the FairFace dataset was explicitly curated to ensure balanced representation across seven major racial categories, including Black, Latino, East Asian, Southeast Asian, Indian, and Middle Eastern populations. This diversity enables models trained on FairFace to demonstrate improved generalization and subgroup fairness, particularly for historically underrepresented groups (Karkkainen and Joo, 2021).

### 3.2 Bias in large language models

Large language models (LLMs) systematically perpetuate and amplify societal stereotypes due to their training on web-scale corpora, as demonstrated by benchmark studies and embedding space analyses (Bender et al., 2021). The Word Embedding Association Test (WEAT) *S*(*X, Y, A, B*) (Caliskan et al., 2017) quantifies these biases by measuring the cosine similarity between target concepts (e.g., male/female names) and attributes (e.g., career/family words), revealing persistent gender and racial associations (Kotek et al., 2023; Cheng et al., 2023).

#### 3.2.1 Evidence grade and provenance

Unless noted otherwise, all bias percentages that follow are *verbatim* from the Parity Benchmark PB-1.1 released by Simpson S. et al. (2024). PB-1.1 contains 350 k English prompt-response pairs covering 14 stereotype categories. Five U.S-based crowd-workers rate each response on a four-point Likert scale; the per-category mean (0–100 %) is the “bias score” we quote here. Limitations: (i) prompts are English-only; (ii) rater demographics are not globally representative; (iii) the scores measure perceived stereotype frequency, not downstream harm. We reproduce the published means and add no new datapoints.

**Figure 3:**
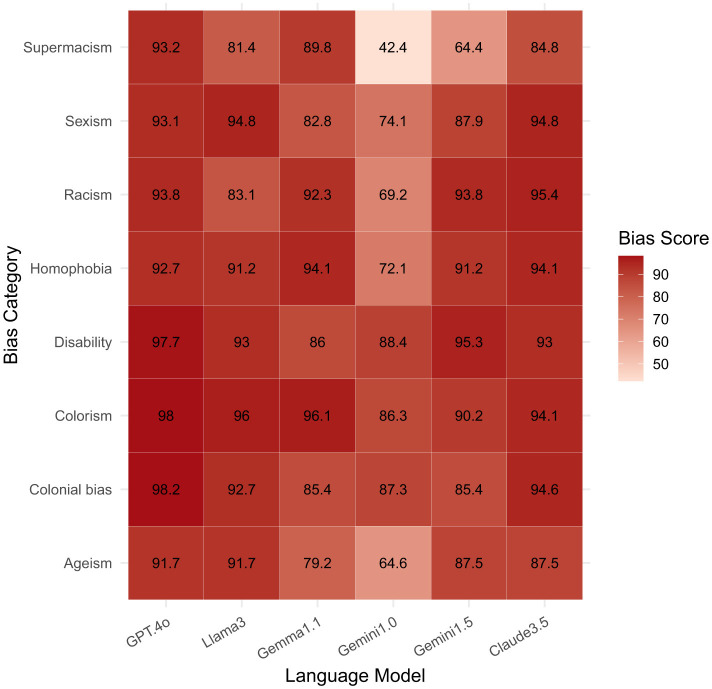
Bias scores (%) across language models, measured using the Parity Benchmark Simpson S. et al. (2024). Higher values indicate stronger model bias. GPT-4o (GPT.4o) consistently shows high bias scores across all categories, notably for colonial bias (98.18%), colorism (98.04%), and disability (97.67%). While Gemini 1.0 also exhibits persistent biases across all types, its scores are relatively lower (42.37–88.37%) compared to other models.

As shown in Figure 3, modern LLMs exhibit striking differences in bias severity across categories. The plotted percentages are drawn directly from the Parity Benchmark (PB-1.1) dataset introduced by Simpson S. et al. (2024), which evaluates language-model responses across protected-category prompts. For example, GPT-4o shows extremely high bias scores for colonial bias (98.18%), colorism (98.04%), and disability (97.67%), while Claude 3.5 exceeds 94% on measures of sexism, racism, and homophobia. In contrast, Gemini 1.0 presents lower benchmark scores across most categories (42.37–88.37%), though it still exhibits measurable bias. These quantitative results reflect disparities in training data and mitigation strategies as well as the persistence of bias in model outputs.

Importantly, benchmark performance does not capture the full extent of representational harm. High-scoring models like GPT-4 and Claude 3.5 may still generate biased or stereotypical language in open-ended text. For instance, Cheng et al. (2023) found that GPT-4 and GPT-3.5 tend to associate terms like *resilient* with Black women and *petite* with Asian women, reinforcing essentialising narratives. Bias thus manifests not only in classification metrics, but also in latent semantic patterns, including dialect preferences and cultural framing. This highlights the need to evaluate models not just for accuracy, but for the broader implications of their linguistic behavior.

#### 3.2.2 Disability-related bias in LLMs

Using the same *Parity Benchmark* PB-1.1 (Simpson S. et al., 2024), the *disability* category shows some of the highest stereotype scores: **GPT-4o 97.7%**, **Claude 3.5 94.1%**, **Llama 3 89.8%**, **Gemini 1.5 87.9 %**, **Gemma 1.1 83.1%**, and **Gemini 1.0 72.1%**. Because a score of 0 % would indicate no stereotypical content, these figures show that disability-related bias is at least as severe as the race- and gender-based disparities discussed above, underscoring the need to look beyond the “usual two” protected attributes.

### 3.3 Bias in algorithmic credit scoring

Algorithmic credit scoring, especially using modern machine learning, is often viewed as a more objective alternative to human decision-making. However, growing evidence shows that these systems frequently replicate, and can even intensify, historical patterns of financial exclusion through feedback effects. This dynamic risks creating a cycle in which individuals from marginalized groups are denied credit or offered worse terms, thereby limiting their ability to build favorable credit histories.

Large-scale studies from the U.S. mortgage market demonstrate that machine learning (ML) models can exacerbate racial disparities in lending outcomes, even when protected attributes such as race or ethnicity are explicitly excluded from model inputs (Bartlett et al., 2022; Fuster et al., 2021). This phenomenon arises from two interrelated mechanisms. First, model flexibility: nonlinear learners (e.g., random forests, gradient boosting, deep neural networks) capture complex interactions among borrower characteristics, leading to greater dispersion in predicted default risk. Compared to simpler models such as logistic regression, the prediction distribution from more flexible models often constitutes a mean-preserving spread, which disproportionately affects applicants whose financial profiles exhibit greater variability, often due to systemic socioeconomic inequities. In particular, Black and Hispanic borrowers are more likely to be placed in the upper tail of the risk distribution and consequently face higher rejection or pricing rates, even when average risk levels remain comparable. Second, proxy reconstruction: even in the absence of explicit racial or ethnic variables, features such as ZIP code, employment history, and income can act as proxies, enabling the model to infer sensitive attributes indirectly. As a result, model outputs risk embedding and amplifying the structural inequalities already present in the training data.

Empirical analyses confirm these patterns. Between 2009 and 2015, ML credit models approved more applicants overall than logistic regression, yet conditional on the same predicted probability of default, Black and Hispanic borrowers were charged higher interest rates and experienced greater variability in credit outcomes compared to White borrowers (Bartlett et al., 2022). Similar dynamics have been observed in other domains, such as insurance underwriting, auto loans, and investment advising, where ostensibly neutral features encode legacies of residential segregation and income stratification. These findings highlight the importance of fairness-aware model design, such as counterfactual fairness representations and equalized-odds constraints, along with rigorous auditing practices and regulatory oversight that treat proxy reconstruction as a form of indirect discrimination.

### 3.4 Healthcare applications

Clinical decision algorithms can only lead to optimal outcomes when grounded in current medical knowledge, yet they have been shown to systematically underperform for certain demographic groups, resulting in disparities in care (Dennstädt et al., 2021). A widely cited example involves healthcare cost prediction models that underestimated the needs of Black patients by using past healthcare expenditures as a proxy for medical necessity (Obermeyer et al., 2019). This approach encoded structural inequalities into risk assessments, as historical spending patterns reflect unequal access to care rather than actual health status.

#### 3.4.1 A simple group-level fairness diagnostic

Let Ŷ∈[0, 1] denote the predicted risk of a binary outcome *Y*∈{0, 1} and let *A*∈{0, 1} mark membership in a protected group (e.g., *A* = 1 for Black patients, *A* = 0 otherwise). Define the *risk-score gap*


$$\begin{array}{c} {\text{Δ}}_{\text{risk}}:=𝔼\left[Ŷ|A=0\right]-𝔼\left[Ŷ|A=1\right]. \end{array}$$ (12)

A non-zero Δ_risk_ flags systematic under- or overestimation of risk for one group relative to the other.

In light of known clinical biases, it is important to consider how AI can assist in improving patient care. As machine learning becomes increasingly involved in health care decisions, assessing algorithmic biases by comparing prediction accuracy across demographic groups is crucial. Once algorithmic bias is uncovered, clinicians and AI must work together to identify the sources of algorithmic bias and improve models through better data collection and model improvements (Chen et al., 2019).

Medical devices used in vascular aging assessment are classified according to risk-based regulatory frameworks, which determine the level of oversight required prior to clinical use. Table 2 summarizes the EU classification system, which includes Class I (low risk), Class IIa and IIb (medium risk), and Class III (high risk) categories (Mayer et al., 2021). For example, non-invasive diagnostic tools such as digital blood pressure monitors, pulse wave velocity sensors, and imaging devices like MRI or ultrasound scanners typically fall under Class IIa. More complex technologies, such as CT/PET scanners and standalone diagnostic software, are classified as Class IIb. Invasive devices like catheters, used for coronary assessments, are considered high risk and placed in Class III.

**Table 2:** EU medical device risk classification (adapted from Mayer et al., 2021).

**Risk level**	**Class**	**Example devices**
High risk	Class III	Implanted devices (e.g., pacemakers, intravascular catheters)
Medium risk	Class IIa and IIb	Diagnostic monitors, standalone software, imaging systems
Low risk	Class I	Non-invasive basic tools (e.g., stethoscopes, thermometers)

These EU classifications are broadly aligned with the regulatory frameworks used in the United States (FDA) and Australia (TGA), which also adopt a three-tiered system based on device risk. While CE marking is required for EU and Australian markets, U.S. regulations involve pathways such as 510(k), *De Novo*, or Premarket Approval (PMA), depending on device risk and novelty. Risk classification further determines requirements for traceability, post-market surveillance, and clinical evaluation (Mayer et al., 2021).

Importantly, while these classifications are essential for ensuring safety, they can inadvertently contribute to bias in device development and deployment. Lower-risk categories typically face fewer regulatory hurdles, potentially limiting the depth of clinical validation across diverse populations. For instance, Class IIa devices may be approved without sufficient evaluation of performance differences by sex, age, or ethnicity. Moreover, the financial and regulatory burden associated with high-risk categories can discourage the development of advanced devices tailored for underrepresented groups. These dynamics underscore the need to incorporate equity considerations into device validation standards across all risk classes to ensure fairness in vascular aging assessments.

### 3.5 Employment tools

AI technologies are increasingly integrated into recruitment workflows, automating tasks such as applicant screening, interview scheduling, and candidate evaluation (Chen, 2023). Table 3 summarizes the key functions of AI-driven recruitment tools, the causes and types of discrimination they may perpetuate, and strategies to mitigate these issues. These tools assess eligibility criteria, analyze candidate expressions, and predict future performance. However, numerous studies have highlighted that AI systems can unintentionally replicate or even exacerbate hiring biases. Such discriminatory outcomes often stem from flawed software design, biased training datasets, or inaccessible user interfaces. Furthermore, users may exploit these systems by manipulating inputs, such as simulating ideal responses in chatbot interviews, to achieve favorable results.

**Table 3:** Summary of AI-driven recruitment functions, causes of discrimination, and mitigation strategies (adapted from interview data).

**Category**	**Examples**
**AI recruitment functions**	• *Sourcing*: Automated application reviews, eligibility assessments, and scoring mechanisms. • *Interview scheduling*: Auto-scheduling, analysis of candidate expressions, and chatbot-based Q&A. • *Selection*: Predicting candidate performance, optimizing compensation packages, and ranking applicants.
**Causes of discrimination**	• *AI software issues*: Bias in algorithmic design, reliance on skewed training data, and poor accessibility for diverse users. • *User behavior*: Insufficient training for recruiters, and deliberate manipulation of chatbot systems by candidates.
**Types of discrimination**	• *Extrinsic factors*: Biases based on gender, nationality, and other observable traits. • *Intrinsic factors*: Discrimination linked to personality traits, cognitive abilities, or communication styles.
**Anti-discrimination measures**	• *Technical tools*: Implementation of fairness-aware algorithms, guidance for inclusive software design, and machine learning fairness constraints. • *Non-technical measures*: Regulatory oversight, AI-specific hiring laws, and independent third-party audits.

Discrimination manifests along both extrinsic (e.g., gender, nationality) and intrinsic (e.g., personality traits, IQ scores) dimensions. To address these concerns, technical solutions such as fairness-aware machine learning and guidance tools are being developed. Non-technical safeguards, including legal oversight, third-party audits, and government regulation, are also essential to ensure ethical deployment of AI in employment decisions.

Amazon's internal recruitment system (2014–2017) systematically penalized résumés that mentioned women's colleges or included verbs more frequently used by female candidates (e.g., volunteered, mentored) (Dastin, 2018). The algorithm had learned to associate such features with lower hiring likelihood, mirroring historical hiring patterns in which men were overwhelmingly preferred.

Algorithmic hiring systems often lack transparency, which hinders efforts to evaluate how models are developed and whether they adhere to anti-discrimination laws. Vendor practices, such as how prediction targets are defined and how de-biasing is applied, can introduce legal and ethical risks, particularly under statutes like the ADA (Raghavan et al., 2020).

Commercial automated speech recognition (ASR) systems exhibit significantly higher word error rates for Black speakers than for White speakers, despite using the same spoken content. This disparity, documented across systems from Apple, IBM, Google, Amazon, and Microsoft, underscores how linguistic technologies can reflect and amplify racial inequalities (Koenecke et al., 2020).

The U.S. Equal Employment Opportunity Commission (EEOC) and the Department of labor released the AI and Inclusive Hiring Framework (2024), which recommends pre-deployment bias audits and transparent, accessible model explanations (Gigante et al., 2024). New York City Local Law 144 requires third-party bias audits and public disclosure of impact ratios before deploying automated employment decision tools. The EU AI Act similarly classifies AI used in labor-related decision-making as high risk, mandating conformity assessments and continuous post-market monitoring.

### 3.6 Criminal justice systems

Risk assessment instruments in criminal justice decision-making have gained wide traction for their promise of data-driven objectivity. Tools such as COMPAS, used in parole, bail, and sentencing determinations, aim to streamline evaluations of an individual's likelihood of recidivism. However, scrutiny reveals systemic inequities in how such models are developed and deployed. ProPublica's investigation of COMPAS, for instance, showed that Black defendants were nearly twice as likely as White defendants to be wrongly categorized as high-risk recidivists, despite comparable actual reoffending rates (Angwin et al., 2016).

Examples were presented in which COMPAS scores labeled individuals with extensive criminal histories as low risk. Such outcomes were attributed to the lack of transparency in the COMPAS system, potentially resulting in unsafe conditions for the public. Even if COMPAS satisfied some reasonable definition of fairness, its lack of transparency raises concerns about procedural fairness, particularly when misclassifications or unexplained discrepancies in risk scores affect individual outcomes. It has been established that COMPAS does not outperform simpler, interpretable models in predicting recidivism. Therefore, the continued use of complex, proprietary models, despite their opacity, cost, and susceptibility to error, has not been justified. The perceived superiority of black-box models has been questioned, as proprietary status does not inherently indicate predictive advantage over publicly available alternatives (Rudin et al., 2020).

This discrepancy reflects unequal false positive rates (FPRs) across protected groups. The following proposition formalizes the corresponding fairness criterion:

Proposition 1. A binary classifier satisfies *false-positive-rate parity* iff


Pr(Ŷ=1∣Y=0,a=0)=Pr(Ŷ=1∣Y=0,a=1).

Proof. By definition, the false positive rate is the probability of a positive prediction given a true negative label, conditional on group membership. The stated equality directly formalizes parity of false positive rates across groups.

In a systematic review of external validation studies on 11 commonly used risk assessment tools, it was found that most investigations reported only the area under the curve (AUC) to describe model performance, without including other critical measures such as false positive and false negative rates or calibration (Fazel et al., 2022). As a result, it has been recommended that researchers prioritize addressing the key methodological limitations identified in prior studies. For jurisdictions considering the adoption of such instruments, independent validation studies should be conducted as part of the implementation process. Predictive performance is to be considered alongside factors such as scalability, transparency, and ethical implications.

Table 4 and the boxed credit-scoring walk-through illustrate how a mathematical audit signal propagates to a concrete governance action and documentation trace.

**Table 4:** End-to-end audit map: from bias family to governance cadence.

**Bias family**	**Primary metric(s)**	**Audit artifact**	**Governance lever**	**Cadence**
Historical / representational	weat, embedding bias	Dataset datasheet	ISO 42001 procurement checklist	Once per corpus
Selection / measurement	ΔTPR, missing-rate heat map	Sampling protocol log	Data-collection Standard Operating Procedure	Quarterly
Algorithmic / optimization	EO, DP, calibration gap	Model card	Regulator filing (e.g. CFPB)	Each retrain
Feedback / emergent	Spectral radius ρ(*J*_*h*_), drift test	Live dashboard	Internal risk-committee minutes	Monthly

## 4 Strategies to address bias

Table 4 links the formal fairness metrics developed in Sections 2–3 to concrete socio-technical controls and documentation artifacts required for continuous governance.

Improving training data remains a foundational strategy for mitigating algorithmic bias. This includes expanding dataset diversity, balancing demographic representation, and developing targeted supplementary datasets for underrepresented groups. Techniques such as stratified data collection, synthetic augmentation, and oversampling can help close representational gaps, though synthetic methods must be carefully designed and validated to avoid reproducing existing biases. A framework for algorithmic auditing has been proposed using a case study of pymetrics, a company that applies machine learning to match job candidates with potential employers (Wilson et al., 2021). The company's approach to fairness has been analyzed in light of ethical guidelines, regulatory obligations, and client requirements. The implementation of adverse impact testing within pymetrics' software has also been examined. Furthermore, the outcomes of an independent audit of the candidate screening tool have been reported. The paper concludes with recommendations on how audits can be designed to remain practical, independent, and constructive, in order to promote greater industry participation in third-party evaluations and to better equip oversight groups in investigating algorithmic systems.

The Datasheets for Datasets framework (Gebru et al., 2021) enhances transparency and accountability in machine learning by providing structured documentation of datasets, including their origins, intended uses, and limitations. This supports informed use, mitigates bias, and promotes reproducibility. Structured dataset documentation supports informed selection and early identification of biases, aligning with emerging research that prioritizes equity-focused data quality assessments to address representational harms upstream in the machine learning pipeline (Gebru et al., 2021; Barocas et al., 2019). Emerging research advocates for proactive, equity-focused data quality assessments early in dataset development to identify and address biases upfront, reducing reliance on complex downstream mitigation efforts.

**Figure 4:**
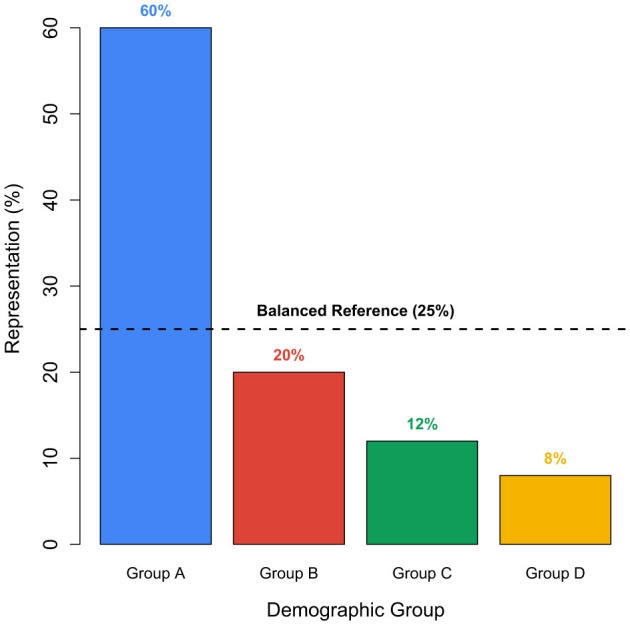
This figure illustrates disparities in demographic representation within a hypothetical training dataset, relative to a balanced benchmark (25% per group). While not a direct measure of bias, it conceptually highlights how imbalanced data can propagate unfairness during model development. Such visualizations support the need for dataset documentation practices as emphasized by Gebru et al. (2021).

Figure 4 provides a hypothetical example demonstrating how demographic groups can be substantially over- or underrepresented in a typical training dataset. In this example, Group A constitutes 60% of the dataset, whereas Groups C and D represent only 12% and 8%, respectively-significantly diverging from the balanced reference level of 25%. While these values are illustrative and not based on empirical data, they underscore the kinds of disparities that can arise during dataset creation. Equity-focused documentation practices, such as datasheets for datasets (Gebru et al., 2021), are intended to highlight and mitigate such imbalances, promoting fairness and transparency in AI development.

### 4.1 Challenges of fairness in machine learning models

The technical fairness literature proposes various mathematical definitions for equity in model development, including demographic parity, equalized odds, and individual fairness. However, implementing these metrics is challenging because multiple fairness criteria are often incompatible and cannot be satisfied simultaneously.

#### 4.1.1 Demographic parity

Ensures similar prediction rates across groups:


P(Ŷ=1∣A=a)=P(Ŷ=1∣A=b) ∀a,b∈Groups

where Ŷ is the predicted outcome, and *A* represents the sensitive attribute (e.g., race, gender).

#### 4.1.2 Equalized odds

Equalized odds requires that a classifier has equal true positive and false positive rates across protected groups:


P(Ŷ=1∣Y=y,A=a)=P(Ŷ=1∣Y=y,A=b) ∀y∈{0,1}

This criterion addresses disparities in error rates and is a central form of group fairness.

#### 4.1.3 Individual fairness

Individual fairness requires that similar individuals receive similar outcomes:


$$d\left({Ŷ}_{i},{Ŷ}_{j}\right)\leq d\left(X_{i},X_{j}\right)\;\forall i,j$$

where *d*(·, ·) is a task-relevant distance metric.

Formal results show that these fairness criteria often conflict, meaning they cannot all hold simultaneously in the same model, especially when the base rates (prevalence of the outcome) across groups differ (Caton and Haas, 2024).

### 4.2 Technical fairness interventions across the ML pipeline

Debiasing techniques, such as adversarial learning, fairness constraints, and preprocessing interventions, offer structured approaches to improve fairness metrics in machine-learning systems. However, their implementation requires careful consideration of both legal requirements and practical limitations, including potential trade-offs with predictive accuracy and model interpretability. These techniques are typically categorized by the stage at which they intervene in the machine-learning pipeline.

Pre-processing methods operate on the input data prior to model training and include strategies such as reweighting, resampling, or transforming features to reduce bias. In-processing techniques embed fairness objectives directly into the model training phase, employing mechanisms such as fairness-aware loss functions or adversarial debiasing. While adversarial approaches can effectively reduce disparities across groups, they may also suppress informative features or degrade model performance, potentially leading to outputs that appear fair but are poorly calibrated or unstable over time. Post-processing methods adjust model predictions after training and include procedures like threshold shifting, output recalibration, or group-specific decision rules (Caton and Haas, 2024; Feldman et al., 2015; Zhang et al., 2018).

While pre- and post-processing methods tend to be more flexible and model-agnostic, in-processing techniques offer tighter integration with the learning process and can yield stronger fairness-performance trade-offs when carefully applied.

Fairness-aware optimization and adversarial learning have already proved useful in practice. For example, adversarial training reduced demographic bias in toxicity-classification tasks on the CIVIL COMMENTS dataset (Zhang et al., 2018). Likewise, fairness-aware credit-scoring models have delivered more equitable outcomes across demographic groups while maintaining accuracy. Table 5 summarizes how these mitigation strategies align with specific bias types.

**Table 5:** How different mitigation strategies address specific types of bias (adapted from Caton and Haas, 2024).

**Bias type**	**Effective mitigation strategies**
Historical / representational	Dataset diversification; participatory data collection
Selection / measurement	Pre-processing (re-weighting, re-labeling); fairness-aware sampling
Algorithmic / optimization	In-processing (adversarial training; fairness constraints)
Feedback loop	Post-processing; dynamic audits; continuous monitoring

Mathematically, common group-fairness criteria include *Demographic Parity (DP)* and *Equalized Odds (EO)*:^1^


$$\begin{array}{l} \text{Demographic parity:}\;\mathrm{Pr}\left({Ŷ}_{\theta }=1|A=0\right)=\\ \;\mathrm{Pr}\left({Ŷ}_{\theta }=1|A=1\right), \end{array}$$ (13)

$$\begin{array}{l} \text{Equalized odds:}\;\mathrm{Pr}\left({Ŷ}_{\theta }=1|Y=y,A=0\right)=\\ \;\mathrm{Pr}\left({Ŷ}_{\theta }=1|Y=y,A=1\right),\\ \;\forall y\in \left\{0,1\right\}. \end{array}$$ (14)

A typical learning objective incorporates these criteria either as constraints or as penalties:


$$\begin{array}{l} \underset{\theta }{\min}L\left({Ŷ}_{\theta },Y\right)\;\text{s.t. Fairness}\left({Ŷ}_{\theta },A\right)\leq \varepsilon , \end{array}$$ (15)

$$\begin{array}{l} \underset{\theta }{\min}L\left({Ŷ}_{\theta },Y\right)+\lambda \text{ Fairness}\left({Ŷ}_{\theta },A\right),\;\lambda \geq 0. \end{array}$$ (16)

**Figure 5:**
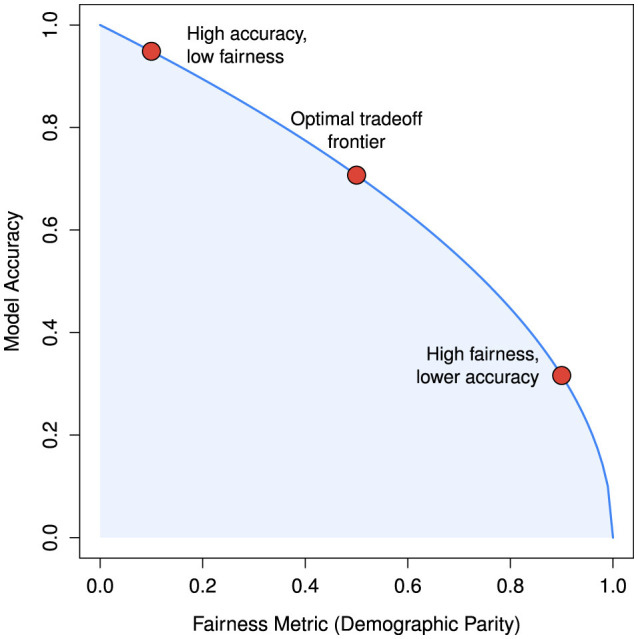
Conceptual illustration of the Pareto frontier showing the tradeoff between fairness and accuracy. The curve is synthetically generated to reflect the general inverse relationship between fairness (measured by demographic parity) and predictive accuracy, and does not represent empirical results.

Figure 5 conceptually illustrates the well-documented tension in machine learning between predictive accuracy and fairness. Models optimized solely for accuracy may achieve high performance at the expense of equitable outcomes. In the extreme case, represented by the top-left corner of the figure (fairness = 0, accuracy = 1), a model attains maximal accuracy only by entirely neglecting fairness constraints, effectively favoring the majority group or those with greater data representation. This idealized scenario mirrors real-world patterns, where optimizing exclusively for accuracy can yield highly performant yet inequitable models. The figure, synthetically generated using demographic parity as the fairness metric, serves as a conceptual visualization and does not represent empirical results. These considerations underscore the ethical and regulatory imperative to balance model performance with fairness across demographic groups, especially in high-stakes applications.

### 4.3 Transparency, governance, and regulatory oversight

Transparent ML practices help stakeholders detect and address bias through interpretable models (e.g., decision trees) or *post-hoc* explanation tools (e.g., LIME, SHAP). Explainable-AI frameworks reveal feature importance and decision boundaries; however, Kaur et al. (2020) warn that poorly designed explanations can foster unwarranted confidence.

Documentation standards such as *Model Cards* (Mitchell et al., 2019) and *Datasheets for Datasets* (Gebru et al., 2021) formalize reporting of model purpose, subgroup performance, and known limitations, enabling practitioners to judge fitness for use.

Regulatory and standardization efforts are increasingly institutionalizing fairness in artificial intelligence systems. The NIST AI Risk Management Framework 1.0 (2023) offers structured guidance for identifying and mitigating AI-related risks, including algorithmic bias, by promoting best practices for trustworthy AI development and deployment (National Institute of Standards and Technology, 2023). Complementing this, ISO/IEC 42001:2023 establishes global requirements for AI management systems, with a focus on lifecycle governance, accountability, and transparency (International Organization for Standardization, 2023a). This is complemented by the AI Governance Alliance: Global Standards for Responsible AI initiative launched by the World Economic Forum in 2025 (World Economic Forum & Accenture, 2025), which emphasizes cross-sector alignment, transparency, and accountability via a multistakeholder governance framework. At the municipal level, New York City Local Law 144 mandates independent third-party bias audits for automated employment decision tools, introducing a legally enforceable mechanism for assessing algorithmic fairness prior to deployment (New York City Council, 2021).

Industry consortia (IEEE, ISO) translate ethical commitments into actionable technical guidelines (Koene et al., 2018; International Organization for Standardization, 2023b). Effective bias mitigation also demands interdisciplinary collaboration that engages social scientists, legal scholars, ethicists, and crucially-affected communities, whose perspectives help ensure that AI systems serve those most vulnerable to harm.

## 5 Application domains and cross-cutting themes

### 5.1 Landscape of high-stakes decision domains

AI-driven decision tools have proliferated most rapidly in five high-stakes arenas, healthcare, criminal justice, finance, education and employment, largely because each offers (i) abundant digital traces, (ii) high expected value per decision, and (iii) strong political pressure for auditability (European Union, 2024). Table 6 summarizes the characteristic data modalities, typical performance targets, and known fairness failure modes for each domain.

**Table 6:** Key characteristics and representative fairness challenges across high-stakes domains.

**Domain**	**Typical data types**	**Main ML task**	**Representative fairness challenge**
Healthcare	EHRs, billing codes, imaging	Risk prediction, diagnosis	Racial bias in comorbidity proxies
Criminal justice	Arrest records, court filings	Recidivism prediction	Unequal FNR/FPR across racial groups
Finance	Credit bureau files, bank transactions	Credit scoring	Proxy discrimination via location features
Education	LMS clickstreams, grades	Dropout forecasting	Amplification of achievement gaps
Employment	Resumés, video interviews	Candidate ranking	Gender bias from historical hires

#### 5.1.1 Healthcare

Clinical risk scoring and diagnostic support systems must balance individual-level accuracy with equitable population-level outcomes. Racial bias commonly emerges from training on billing-code proxies for disease burden (Obermeyer et al., 2019).

#### 5.1.2 Criminal justice

Recidivism prediction instruments such as COMPAS illustrate the tension between predictive parity and equalized odds. Disparate error rates along racial lines have triggered landmark policy debates (Angwin et al., 2016).

#### 5.1.3 Finance

Credit-scoring models increasingly rely on non-traditional features (e.g. social-network signals), complicating compliance with fair-lending regulation, where seminal work highlights proxy-based discrimination even after legally protected attributes are removed (Bartlett et al., 2022).

#### 5.1.4 Education

Learning analytics platforms increasingly inform interventions such as tutoring, grading support, and course recommendations. While these systems can personalize learning, bias in training data and modeling choices can inadvertently widen existing achievement gaps (Holstein et al., 2019).

#### 5.1.5 Employment

Resume-screening and interview-ranking systems have been shown to inherit gender and age biases from historical hiring data (Raghavan et al., 2020; Wilson et al., 2021). Transparency and auditability mandates, such as New York City's Local Law 144, now provide natural test-beds for governance-focused interventions (New York City Council, 2021).

These domain snapshots motivate the need for a unifying analytical scaffold-provided in this mini-review by the four-family taxonomy introduced in Section 2.

### 5.2 Mapping the four families to domain-specific challenges

Having outlined the landscape, we now illustrate how each fairness family (Data, Algorithm, Interface, Governance) addresses the concrete failure modes surfaced in Section 5.1.

#### 5.2.1 Family 1-Data-centric interventions

Healthcare: In healthcare, reweighting electronic claims data using causal adjustment has been shown to reduce racial bias in risk scores by approximately 23% Chen et al. (2019). In employment contexts, synthetic minority oversampling can narrow gender disparities in résumé ranking without compromising overall predictive precision De-Arteaga et al. (2019).

#### 5.2.2 Family 2–Algorithm-level constraints

**Criminal justice:** imposing fairness constraints such as *predictive equality* (equalizing error rates across groups) on learned risk scores can substantially reduce disparity with only modest utility loss (Corbett-Davies et al., 2017; Pleiss et al., 2017).

**Finance:** adversarial and constrained-optimization approaches achieve regulatory parity targets in credit scoring while maintaining strong ranking performance, as measured by the Area Under the ROC Curve (AUC) and the Kolmogorov–Smirnov statistic (KS) (Zhang et al., 2018; Agarwal et al., 2018; Kozodoi et al., 2022; Madras et al., 2018).

#### 5.2.3 Family 3–Interface-level mediation

**Education:** explanation and interface tooling (e.g., dashboards, counterfactual-style widgets) can help practitioners interpret predictions and adopt systems more appropriately Holstein et al. (2019). **Healthcare:** clinician-facing decision aids require careful trust calibration; interfaces should surface uncertainty and model limits to avoid over-reliance and the propagation of underlying biases Obermeyer et al. (2019); Chen et al. (2019).

#### 5.2.4 Family 4–Governance frameworks

**Finance & Employment:** transparency and auditability mandates, such as New York City's Local Law 144, have institutionalized algorithmic impact assessments (AIAs) and periodic bias audits as part of model governance (New York City Council, 2021; Wilson et al., 2021). **Criminal justice:** frameworks for accountability increasingly emphasize independent oversight and documentation, including bias evaluation protocols aligned with international standards such as ISO/IEC 42001 and the NIST AI Risk Management Framework International Organization for Standardization (2023a).

Table 7 provides a concise alignment matrix of families × domains, highlighting empirically demonstrated performance and equity improvements. As shown in Table 5, pre-processing, in-processing, and post-processing techniques target different kinds of group-level disparities.

**Table 7:** Family-level mitigation levers aligned to each domain.

**Domain**	**Data**	**Algorithm**	**Interface**	**Governance**
Healthcare	Re-weight claims; augment cohorts	Equalized-odds risk models	Clinician dashboards	FDA post-market monitoring
Criminal justice	Audit and repair arrest data	Parity-constrained trees	Plain-text risk notes	Community oversight boards
Finance	Debias credit files	Adversarial scoring / regularization	Loan-officer explainers	Fair-lending audits
Education	Balance cohorts / clickstreams	Fair dropout models	Student-facing recommenders	Impact reviews
Employment	Diverse resumé corpora	Bias-mitigated ranking	Accessible applicant chatbots	NYC Local Law 144 audits

The matrix reveals complementarities: data-level fixes often enable more effective algorithmic constraints, while governance structures create the long-term incentives necessary to maintain interface and modeling choices that favor equity.

### 5.3 AI-for-social-good as a cross-cutting lens

The The AI-for-Social-Good (AI4SG) agenda seeks to marshal AI techniques toward public-interest goals such as the U.N. Sustainable Development Goals, grounded in ethical principles of a “good AI society” as articulated in the AI4People framework (Floridi et al., 2018). Because such projects often operate in high-stakes, resource-constrained settings, fairness becomes inseparable from safety, accountability, and long-term sustainability, challenges which AI4People frames through principles like justice and explicability. The four-family taxonomy (Data / Algorithm / Interface / Governance) thus offers a structured lens for diagnosing failures and guiding design in AI4SG initiatives.

#### 5.3.1 Family 1–Data-centric interventions

Many AI4SG deployments begin with skewed or incomplete datasets that mirror existing structural inequities and propagate “data cascades” in high-stakes settings (Buolamwini and Gebru, 2018; Karkkainen and Joo, 2021). Empirical work shows that under-representation of demographic groups produces systematic performance gaps, especially in vision and language tasks (Buolamwini and Gebru, 2018; Karkkainen and Joo, 2021). Family 1 remedies therefore emphasize *pre-deployment* data work: targeted sampling and augmentation, dataset documentation (Datasheets) and transparent model reporting (Model Cards), which improve coverage and help surface residual risks before deployment (Gebru et al., 2021; Mitchell et al., 2019).

#### 5.3.2 Family 2–Algorithm-level fairness constraints

Fairness-aware optimization techniques directly embed ethical constraints into model training. In high-stakes domains such as healthcare and finance, imposing fairness metrics like *equalized odds* or *predictive equality* has been shown to narrow disparities in error rates across demographic groups with minimal performance loss Corbett-Davies et al. (2017); Pleiss et al. (2017); Obermeyer et al. (2019); Bartlett et al. (2022). Such algorithm-level constraints translate normative fairness principles into operational learning objectives, ensuring that improvements in predictive accuracy do not exacerbate inequity.

#### 5.3.3 Family 3–Human-facing interface adaptations

Human-AI interaction is itself a locus of bias. Well-designed interfaces can calibrate user trust and improve equitable use of model outputs. In education and healthcare, explanation dashboards and clinician-facing visual aids help users interpret model recommendations, reduce over-reliance, and foster accountability (Holstein et al., 2019; Chen et al., 2019; Obermeyer et al., 2019). Conversely, poorly designed explanation tools can mislead end-users or amplify confirmation bias (Kaur et al., 2020). Family 3 interventions therefore focus on transparency and interpretability artifacts that promote fairness through informed human judgment.

#### 5.3.4 Family 4–Governance and oversight mechanisms

Long-term fairness depends on institutional accountability. Regulatory frameworks such as the NIST AI Risk Management Framework and ISO/IEC 42001 establish governance structures for continuous auditing and documentation (National Institute of Standards and Technology, 2023). Municipal policies like New York City's Local Law 144 require independent bias audits before deploying automated employment tools (New York City Council, 2021), while cross-sector standards and participatory oversight boards (Wilson et al., 2021; Koene et al., 2018) institutionalize fairness as an ongoing governance obligation rather than a one-off technical correction.

#### 5.3.5 Synthesis

Across the AI-for-Social-Good landscape, fairness manifests through interconnected layers: (1) *data-centric remedies* improve representational equity; (2) *algorithmic constraints* formalize ethical criteria within learning objectives; (3) *interface adaptations* enhance interpretability and trust; and (4) *governance mechanisms* sustain accountability through audits and standards (Floridi et al., 2018; Barocas et al., 2019). Together, these layers form a virtuous socio-technical cycle in which improvements at one level reinforce progress at others.

## 6 Discussion and future directions

Addressing AI bias requires navigating complex trade-offs between competing values. Optimizing one fairness metric often undermines others, and interventions can reduce predictive accuracy or increase computational costs. These challenges demand explicit value judgments to determine acceptable compromises within specific contexts. The tension between group-based and individual fairness metrics reflects deeper philosophical debates about equity versus equality, necessitating transparent deliberation and contextual understanding.

Purely technical solutions often fall short by abstracting away the structural and institutional factors underlying algorithmic harms. Such approaches risk perpetuating the status quo instead of challenging and transforming it (Abebe et al., 2020). Sociotechnical frameworks provide a more comprehensive response by recognizing the interplay between algorithms, social systems, and institutional practices. Recent advancements include causal fairness models, distributive justice principles, and contestability mechanisms that aim to shift power dynamics and actively involve affected individuals in the design, oversight, and evaluation of systems.

To complement technical and data-centric interventions, we propose an integrated socio-technical framework that embeds fairness considerations throughout the entire AI lifecycle. This framework underscores the importance of assembling diverse teams, fostering community engagement, implementing iterative feedback mechanisms, and conducting continuous auditing from the design phase to final deployment.

**Figure 6:**
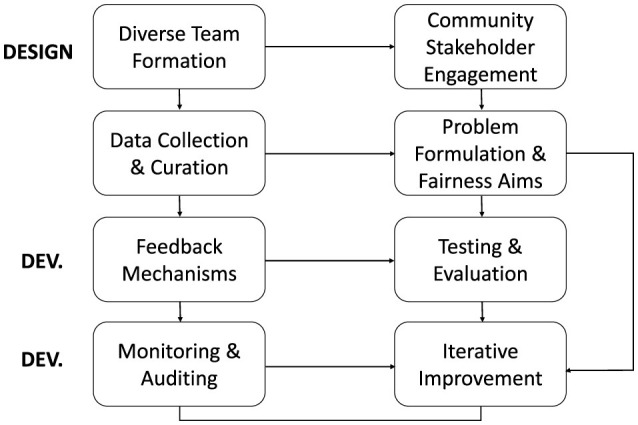
Integrated socio-technical framework for responsible AI development. The framework outlines key stages across the AI lifecycle, including design, development, and deployment (Dev.) phases, spanning problem formulation, data collection, testing, monitoring, and iterative improvement, while emphasizing community engagement, stakeholder inclusion, and fairness considerations.

Figure 6 illustrates the key stages, processes, and feedback loops essential for developing responsible AI systems. It highlights the integration of socio-technical considerations, such as stakeholder engagement, fairness-aware design, and iterative refinement.

Participatory and intersectional approaches are increasingly adopted to address how intersecting identity dimensions (e.g., race, gender, class) influence algorithmic harms. Longitudinal studies further illuminate the societal impacts of AI systems over time. Frameworks such as those proposed by Selbst et al. (2019) highlight the importance of sociotechnical context, institutional structures, and evolving power dynamics in the evaluation of algorithmic interventions.

Effective bias mitigation must align technical solutions with legal non-discrimination standards. Policy frameworks are increasingly requiring algorithmic impact assessments for high-risk deployments, although standardized methodologies are still evolving. Additionally, procurement policies and industry standards, such as ISO 42001, incentivise responsible AI development practices (Koene et al., 2018; International Organization for Standardization, 2023a). These alignments between technical, legal, and institutional efforts are essential for creating equitable and accountable AI systems.

## 7 Conclusion

The challenge of mitigating bias in AI systems represents a critical frontier in both computer science and social science research, demanding solutions that bridge technical innovation with ethical governance. This letter demonstrates that algorithmic bias manifests through three primary channels: structural mechanisms revealed through causal inference frameworks, measurement artifacts embedded in data collection protocols, and dynamical amplification via sociotechnical feedback loops. These insights fundamentally reshape conventional approaches to fairness by moving beyond static correlational analyses toward models that capture the temporal and systemic nature of discrimination in automated systems.

Operationalizing fairness in real-world systems exposes structural tensions that cannot be resolved through technical solutions alone. The impossibility of simultaneously satisfying competing fairness criteria, coupled with context-dependent trade-offs between individual and group equity, necessitates governance frameworks capable of adaptive regulation. Such frameworks must integrate continuous auditing protocols with participatory design methodologies, recognizing that, as highlighted in Figure 6, bias mitigation is an ongoing process requiring feedback loops, community oversight, and sustained institutional accountability. The development of institutional review boards for production AI systems, modeled after biomedical research oversight but adapted for computational contexts, emerges as a promising direction for ensuring accountability.

At stake is the equitable distribution of access to society's most consequential resources, a concern that elevates algorithmic fairness from academic exercise to urgent civil rights imperative. labor markets increasingly rely on hiring algorithms and productivity monitoring systems, while essential services from mortgage approvals to healthcare triage deploy predictive tools with life-altering consequences. These systems risk institutionalizing historical inequities through three compounding pathways. Statistical discrimination can proxy protected attributes, measurement bias may distort the characteristics of marginalized groups, and feedback loops can amplify initial disparities over time. Documented cases in facial recognition, criminal risk assessment, and targeted advertising demonstrate how technical systems can silently harden societal divisions.

Progress requires parallel advances across four interconnected domains. First, temporal fairness metrics must account for how biases evolve in deployed systems, moving beyond snapshot evaluations. Second, participatory design practices should center affected communities in system development, resisting the tendency toward purely technical solutionism. Third, improved bias propagation models must quantify how errors compound across interconnected decision points. Fourth, institutional governance mechanisms need development to provide ongoing oversight of production systems. These directions collectively point toward a reconceptualization of AI development as an explicitly values-driven process that embraces both mathematical precision and sociological insight.

Ultimately, fair AI is neither a purely mathematical pursuit nor a purely political mandate; it is a continuous, interdisciplinary commitment. By coupling rigorous causal analysis with participatory governance and by viewing deployment as the start-not the end-of accountability, we can transform algorithmic systems from vectors of inequity into instruments of shared social progress.

## Footnotes

1 DP requires equal positive prediction rates across groups, while EO additionally conditions on the true outcome label.
