# Supplementary material for: Bias in AI systems: integrating formal and socio-technical approaches
Source: Front Big Data. 2026 Jan 8;8:1686452. doi: 10.3389/fdata.2025.1686452 (PMC12823528; doi:10.3389/fdata.2025.1686452)
Supplement: Supplementary file 1 [file Data_Sheet_1.pdf]

1 SUPPLEMENTARY MATERIALS

LEMMA 1. *Let $R_1 = \widehat{R}_{\mathcal{M}_1}(X)$ be the default-risk predictions returned by a baseline logistic regression model, and $R_2 = \widehat{R}_{\mathcal{M}_2}(X)$ be the predictions of a strictly more flexible learner trained on the* same *feature set X. Assume:*

A1 ***Calibration in the large:***

$$\mathbb{E}[R_1] = \mathbb{E}[R_2] = \mathbb{E}[Y], \quad \textit{where } Y \in \{0,1\}.$$

A2 ***Smaller mean-squared-error (MSE):***

$$\mathrm{MSE}(R_2) := \mathbb{E}\Big[(R_2 - Y)^2\Big] < \mathbb{E}\Big[(R_1 - Y)^2\Big] =: \mathrm{MSE}(R_1).$$

A3 ***Model-nesting (refinement) property:***

$$\mathbb{E}[R_2 \mid R_1] = R_1, \qquad \textit{i.e., } R_1 \textit{ is a measurable coarsening of } R_2.$$

Assumption 3 (A3) is satisfied, for instance, when:

- *$R_1 = g(\beta^\top X)$ depends only on the single index $\eta = \beta^\top X$, and*
- *R_2 is any consistent estimator of $\mathbb{E}[Y \mid X]$.*

Then, by the law of iterated expectation:

$$\mathbb{E}[R_2 \mid \eta] = \mathbb{E}[Y \mid \eta] = g(\eta) = R_1.$$

Then the prediction distribution of the flexible learner is a mean-preserving spread *of the logistic-regression prediction:*

$$R_2 \succ_{MPS} R_1.$$

FULL PROOF. *MSE Decomposition.* Let $p(X) = \mathbb{E}[Y \mid X]$ be the true conditional default probability. Since $Y \sim \mathrm{Bernoulli}(p(X))$, the irreducible (Bayes) risk is

$$\sigma^2(X) = p(X)(1 - p(X)).$$

Then for any predictor $R = R(X)$,

$$\mathrm{MSE}(R) = \mathbb{E}[(R - p)^2] + \mathbb{E}[\sigma^2(X)].$$

Since the second term is constant across models, Assumption A2 implies:

$$\mathbb{E}[(R_2 - p)^2] < \mathbb{E}[(R_1 - p)^2]. \tag{1}$$

Mean-preserving Spread. Let $Z := R_2 - R_1$. By Assumption A3,

$$\mathbb{E}[Z \mid R_1] = \mathbb{E}[R_2 \mid R_1] - R_1 = 0.$$

From Assumption A1, $\mathbb{E}[R_1] = \mathbb{E}[R_2]$, so $\mathbb{E}[Z] = 0$. Thus, one can write:

$$R_2 = R_1 + Z, \quad \text{where} \quad \mathbb{E}[Z \mid R_1] = 0. \tag{2}$$

This is the defining property of a mean-preserving spread (MPS) in the sense of Rothschild and Stiglitz (1970). Moreover, since $\text{MSE}(R_2) < \text{MSE}(R_1)$, one must have $R_2 \not\equiv R_1$, hence $Z \not\equiv 0$, i.e., $\mathbb{P}(Z \neq 0) > 0$.

Variance Increase (as a consequence). It follows that:

$$\mathbb{V}(R_2) = \mathbb{V}(R_1 + Z) = \mathbb{V}(R_1) + \mathbb{V}(Z) + 2\mathbb{C}\rtimes\gtrapprox(R_1, Z).$$

From (2) $\mathbb{E}[Z \mid R_1] = 0$ implies:

$$\mathbb{C}\rtimes\gtrapprox(R_1, Z) = \mathbb{E}[R_1 Z] = \mathbb{E}\Big[R_1 \cdot \mathbb{E}[Z \mid R_1]\Big]$$

Hence,

$$\mathbb{V}(R_2) = \mathbb{V}(R_1) + \mathbb{V}(Z) > \mathbb{V}(R_1),$$

since $\mathbb{V}(Z) > 0$ by non-degeneracy.

Conclusion. Combining (2) with Assumption A1, we conclude that R_2 is a non-trivial mean-preserving spread of R_1. Therefore,

$$R_2 \succ_{\text{MPS}} R_1.$$

PROOF. A random variable X is an MPS of Z if and only if it has the same mean and can be written as

$$X = Z + \varepsilon, \qquad \mathbb{E}[\varepsilon \mid Z] = 0, \quad \mathbb{P}(\varepsilon \neq 0) > 0.$$

In line with standard results in risk theory, the orthogonality condition makes X a mean-zero variation of Z, and non-degeneracy ensures that the variation is non-trivial.

Step 1 - Produce The Orthogonal Shake: Set $Z := R_2 - R_1$ and note from (A3) that $\mathbb{E}[Z \mid R_1] = \mathbb{E}[R_2 \mid R_1] - R_1 = 0$. Hence we already have the representation

$$R_2 = R_1 + Z$$

with the desired orthogonality.

Step 2 - Verify Equal Means: Assumption (A1) gives

$$\mathbb{E}[R_1] = \mathbb{E}[R_2],$$

so $\mathbb{E}[Z] = 0$.

Step 3 - Show The Shake is Non-trivial: Write the usual bias-variance decomposition around the Bayes probability $p(X) := \mathbb{E}[Y \mid X]$:

$$\text{MSE}(R) = \underbrace{\mathbb{E}[(R-p)^2]}_{\text{reducible error}} + \underbrace{\mathbb{E}[p(1-p)]}_{\text{Bayes risk}}.$$

The second term is model-independent. Thus, (A2) implies

$$\mathbb{E}[(R_2 - p)^2] < \mathbb{E}[(R_1 - p)^2].$$

If $Z \equiv 0$ a.s. (i.e., R_2 and R_1 were identical), both reducible errors would coincide, leading to a contradiction. Therefore, $\mathbb{P}(Z \neq 0) > 0$.

Conclude: Steps 1-3 establish exactly the three bullet points in the definition of an MPS, so $R_2 \succ_{\text{MPS}} R_1$.

COROLLARY 1. *The refinement property (Assumption 3) holds in any setting where the simpler model is a function of a lower-dimensional summary statistic $T(X)$, and the flexible model provides a consistent estimate of the conditional expectation $\mathbb{E}[Y \mid X]$. For instance, logistic regression depends only on the linear predictor $\beta^\top X$, whereas more flexible models such as gradient boosting, random forests, or neural networks utilise the full covariate vector X. The decomposition shows that a reduction in mean squared error achieved by a more flexible model necessarily entails an increase in predictive variance. The lemma formalises this trade-off using the concept of a mean-preserving spread, which reflects a form of second-order stochastic dominance.*

COROLLARY 2. *A classical implication of $R_2 \succ_{\text{MPS}} R_1$ is that for any strictly convex and increasing function ϕ, we have:*

$$c\mathbb{E}[\phi(R_2)] > \mathbb{E}[\phi(R_1)].$$

Equivalently, for any strictly concave and increasing utility function u, one obtains:

$$\mathbb{E}[u(R_2)] < \mathbb{E}[u(R_1)],$$

unless R_2 and R_1 are almost surely identical.

In economic terms, a (risk-loving) agent with convex preferences would strictly prefer the more spread-out prediction R_2, whereas a (risk-averse) agent with concave utility would prefer the less spread-out variable R_1.

In the predictive, modelling context, one might interpret this as follows: although both models share the same global mean default risk, the more flexible model $\mathcal{M}_2$ produces larger dispersion, a refined partitioning, that typically allows for more accurate discrimination among different sub-populations.

PROPOSITION 1 (Demographic parity ⇒ zero risk-gap). *If the predictor satisfies* demographic parity *(DP),*

$$\mathbb{E}[\hat{Y} \mid A = a] = \mathbb{E}[\hat{Y}] \quad \textit{for every } a \in \{0, 1\}, \tag{3}$$

then $\Delta_{\text{risk}} = 0$.

PROOF. Apply (3) to each conditional mean:

$$\mathbb{E}[\hat{Y} \mid A = 0] = \mathbb{E}[\hat{Y}] = \mathbb{E}[\hat{Y} \mid A = 1].$$

Subtracting the second equality from the first yields $\Delta_{\text{risk}} = 0$.

COROLLARY 3 (Limitations of the risk-score gap).

(a) $\Delta_{\text{risk}} = 0$ *is* not *sufficient for demographic parity. It only guarantees equal* average *scores between the two groups appearing in (12); DP, by contrast, requires* $\mathbb{E}[\hat{Y} \mid A = a] = \mathbb{E}[\hat{Y}]$ *for* every *realization of* A *(including multi-category or continuous sensitive attributes).*

(b) Even when demographic parity can be achieved, it is often clinically undesirable, because underlying medical need legitimately varies across demographic lines. In such cases, fairness notions that equalise error rates*, e.g.,* equalised odds *or* equal opportunity*, may be more appropriate. Nevertheless, the simple statistic* Δ_{risk} *remains a useful first-pass diagnostic for detecting glaring allocation gaps.*

PROOF. (a) Construct a counterexample. Let $A \sim \text{Bernoulli}(\frac{1}{2})$ and draw $\hat{Y} \mid A = a \sim \text{Uniform}(0, 1)$ if $a = 0$ and $\hat{Y} \mid A = a \sim \text{Uniform}(0, 2)$ if $a = 1$. Rescale the second distribution by a factor of $\frac{1}{2}$ so that both groups have mean $\frac{1}{2}$; then $\Delta_{\text{risk}} = 0$ but $\mathbb{E}[\hat{Y} \mid A = 1] \neq \mathbb{E}[\hat{Y}]$, violating DP.

(b) The claim is conceptual: when disease prevalence differs by demographic group, forcing identical $\mathbb{E}[\hat{Y} \mid A]$ leads either to under-treatment of the high-risk group or over-treatment of the low-risk group. Criteria that match false-positive or false-negative rates avoid this pitfall, yet the presence of a large Δ_{risk} still signals a potential fairness problem.

1.1 Worked numerical example on the credit-scoring data set

This subsection reproduces, step by step, the full sequence of calculations described in Section 2. The public data set is the German-Credit corpus (UCI / Hugging Face mirror). A companion Jupyter notebook with exact code is available in the project artefact bundle.

Stage 1: dataset audit.

- Divergence B_{data} (Eq. (2)): 0.067 nats (90 % CI 0.067–0.067).
- Selection ratio R_{sel} (Eq. (4)): 0.90, 5th-95th percentile [0.83, 0.97].
- Label bias B_{label} (Eq. (5)): −7.5 percentage points for the protected group (90 % CI [−12.7, −2.1]; negative = disadvantage).

Note: The expressions Eq. (2), Eq. (4), and Eq. (5) refer to equation numbers in the **main manuscript**.

Empirical risk minimization (ERM).

We use ERM to denote the standard supervised training objective that minimises average predictive loss over the data:

$$\hat{\theta}_{\text{ERM}} \in \arg\min_{\theta} \frac{1}{n} \sum_{i=1}^{n} \ell\Big(f_\theta(x_i),\, y_i\Big). \tag{4}$$

This objective optimises accuracy alone and does not enforce group-fairness criteria such as demographic parity or equalised odds. When a fairness penalty $\lambda\,\Phi(\theta)$ is added to the ERM objective

(cf. Eq. (16)), we refer to *fairness-regularized ERM*:

$$\hat{\theta}_{\text{fair}} \in \arg\min_{\theta} \; \frac{1}{n}\sum_{i=1}^{n} \ell\Big(f_\theta(x_i),\, y_i\Big) \;+\; \lambda\,\Phi(\theta). \tag{5}$$

This mirrors common formulations used in reductions-based and in-processing approaches for fair classification.

Stage 2: model training.

A logistic-regression baseline is compared against a gradient-boosted tree with an equalised-odds penalty $\lambda = 10^{-2}$ (cf. Eq. (16)). Ten-fold cross-validation yields

Model	AUROC	ΔTPR (protected–reference)
Logistic (no constraint)	0.73	−0.084
XGBoost + EO penalty	0.78	−0.012

Note. AUROC = area under the ROC curve; TPR = true positive rate. The fairness penalty λ enforces the equalised-odds constraint.

Stage 3: deployment monitoring.

The system is embedded in the feedback loop of Eq. (8). Empirical estimation of the Jacobian gives $\rho\Big(J_h\Big) = 0.94 < 1$, so the loop is locally stable. Monthly drift tests detect no significant change in ΔTPR over six months.

End-to-end walk-through: credit scoring → governance action

1 **Audit.** Monthly drift test shows $\Delta\text{TPR} = -0.042$ for the protected group (>3 pp weaker than the alert threshold).

2 **Root cause.** Closed-loop Jacobian (9) gives $\rho(J_h) = 1.07 > 1$; error is being *amplified.*

3 **Governance trigger.** Internal policy says ”if $\rho(J_h) > 1$ for two consecutive months $\Rightarrow$ send a remediation plan to the Fair-Lending Committee”.

4. **Technical fix.** Committee accepts switching the scorecard to the equalised-odds–penalised XGBoost ($\lambda = 10^{-2}$) already developed in Sec. 3.3. Re-training reduces $\rho(J_h)$ to 0.94 and ΔTPR to −0.012.

5 **Documentation.** Model card and committee minutes are uploaded to the governance repository, satisfying ISO 42001 traceability.
